# DNA methylation profiles in individuals with rare, atypical 7q11.23 CNVs correlate with *GTF2I* and *GTF2IRD1* copy number

**DOI:** 10.1038/s41525-023-00368-7

**Published:** 2023-09-14

**Authors:** Emma Strong, Carolyn B. Mervis, Elaine Tam, Colleen A. Morris, Bonita P. Klein-Tasman, Shelley L. Velleman, Lucy R. Osborne

**Affiliations:** 1 Department of Molecular Genetics, University of Toronto, Toronto, ON Canada; 2 Department of Psychological and Brain Sciences, University of Louisville, Louisville, KY USA; 3 Department of Medicine, University of Toronto, Toronto, ON Canada; 4 Department of Pediatrics, Kirk Kerkorian School of Medicine at University of Nevada Las Vegas, Las Vegas, NV USA; 5 Department of Psychology, University of Wisconsin-Milwaukee, Milwaukee, WI USA; 6 Department of Communication Sciences and Disorders, University of Vermont, Burlington, VT USA; 7 Departments of Medicine and Molecular Genetics, University of Toronto, Toronto, ON Canada; 8 Present Address: Division of Genome Diagnostics, Department of Pathology and Laboratory Medicine, BC Children’s and Women’s Hospital, Vancouver, BC Canada

**Keywords:** DNA methylation, Molecular medicine

## Abstract

Williams-Beuren syndrome (WBS) and 7q11.23 duplication syndrome (Dup7) are rare neurodevelopmental disorders caused by deletion and duplication of a 1.5 Mb region that includes at least five genes with a known role in epigenetic regulation. We have shown that CNV of this chromosome segment causes dose-dependent, genome-wide changes in DNA methylation, but the specific genes driving these changes are unknown. We measured genome-wide whole blood DNA methylation in six participants with atypical CNV of 7q11.23 (three with deletions and three with duplications) using the Illumina HumanMethylation450k array and compared their profiles with those from groups of individuals with classic WBS or classic Dup7 and with typically developing (TD) controls. Across the top 1000 most variable positions we found that only the atypical rearrangements that changed the copy number of *GTF2IRD1* and/or *GTF2I* (coding for the TFII-IRD1 and TFII-I proteins) clustered with their respective syndromic cohorts. This finding was supported by results from hierarchical clustering across a selection of differentially methylated CpGs, in addition to pyrosequencing validation. These findings suggest that CNV of the *GTF2I* genes at the telomeric end of the 7q11.23 interval is a key contributor to the large changes in DNA methylation that are seen in blood DNA from our WBS and Dup7 cohorts, compared to TD controls. Our findings suggest that members of the TFII-I protein family are involved in epigenetic processes that alter DNA methylation on a genome-wide level.

## Introduction

Williams-Beuren syndrome (WBS, MIM: 194050)^1^ and 7q11.23 duplication syndrome (Dup7, MIM: 609757)^2^ are two rare neurodevelopmental disorders that arise by copy number variation (CNV) (deletion or duplication, respectively) of a 1.5 Mb region on chromosome 7q11.23^3^. The reciprocal nature of these CNVs provides the opportunity to study gene dosage effects on neurodevelopmental phenotypes and molecular pathways. This has been facilitated by the identification of individuals with small, atypical deletion or duplication at 7q11.23, who have aided in understanding the contribution of specific sub-regions to the phenotypes of these disorders. Although rare, studies of individuals with atypical rearrangements over the last 20 years have highlighted the importance of genes at the telomeric end of 7q11.23 in contributing to aspects of the WBS cognitive profile^4–18^.

Previous work by our group identified striking, symmetrical, dose-dependent changes in DNA methylation of peripheral blood cells from children with classic WBS and classic Dup7, compared to typically developing (TD) controls^19^. This unique finding implied that one or more genes within the 7q11.23 CNV contributes to DNA methylation, such that loss or gain results in perturbed DNA methylation.

There are several compelling candidates for effects on DNA methylation within the common 7q11.23 CNV, based on known or predicted function. Toward the centromeric end, *BAZ1B* codes for a component of the ISWI-family of ATP-dependent chromatin remodeling complexes^20^ with homozygous loss of this gene impacting heterochromatin formation^21^. In the middle of the region, *BCL7B* codes for a subunit of the ATP-dependent SWI/SNF chromatin remodeling complex^22^, and *BUD23* for a protein containing S-adenosyl-l-methionine binding and methyltransferase domains that has been implicated in H3K4 methylation and rRNA N7-methylation^23–25^. At the most telomeric end, the general transcription factor genes *GTF2I* and *GTF2IRD1* code for proteins TFII-I and TFII-IRD1 respectively, both of which have been shown to interact with different chromatin remodeling complexes or proteins, including HDAC3^26^. TFII-I has been shown to interact with several additional chromatin remodeling complexes including HDAC1, HDAC2, LSD1, and components of the polycomb repressive complex-2 (PRC2)^27–29^.

To aid in isolating specific genes that contribute to altered DNA methylation in individuals with 7q11.23 CNV, we identified six individuals with rare, atypical deletions or duplications of 7q11.23 that in combination, variably affect the centromeric and telomeric genes associated with epigenetic mechanisms (Table 1). Our goal was to establish whether genome-wide methylation analysis of DNA from these rare individuals could aid in defining the genes at 7q11.23 most likely contributing to aberrant DNA methylation. To this end, we generated genome-wide peripheral blood DNA methylation profiles from these individuals with atypical rearrangements and compared them to previously published profiles of individuals with classic WBS, classic Dup7, and TD controls^19^.

**Table 1:** Atypical 7q11.23 Copy Number Variants (CNVs).

Participant	CNV	Start	End	CNV size	Array	Genes within CNV
Atyp Del1	Loss	74,304,574	74,719,013	414,439 bp	CGH + SNP (GenomeDx)	CLIP2^a^; GTF2IRD1; GTF2I^b^
Atyp Del2	Loss	74,023,916	74,731,820	707,904 bp	SNP Array 6.0 (Affymetrix)	ELN^a^; LIMK1; EIF4H; MIR590; LAT2; RFC2; CLIP2; GTF2IRD1; GTF2I^b^
Atyp Del3	Loss	73,330,491	73,813,707	483,216 bp	CMA + HR + SNP v9.1.1 (Agilent)	FKBP6; FZD9; BAZ1B; BCL7B; TBL2; MLXIPL; VPS37D; DNAJC30; BUD23; STX1A; MIR4284; ABHD11-AS1; ABHD11; CLDN3; CLDN4
Atyp Dup1	Gain	74,002,716	74,232,208	229,492 bp	CGH + SNP 4x180K (Agilent)	ELN; LIMK1; EIF4H; MIR590; LAT2; RFC2^c^
Atyp Dup2	Gain	73,326,323	73,944,794	618,471 bp	OmniExpress PIs (Illumina)	FKBP6; FZD9; BAZ1B; BCL7B; TBL2; MLXIPL; VPS37D; DNAJC30; BUD23; STX1A; MIR4284; ABHD11-AS1; ABHD11; CLDN3; CLDN4; METTL27; TMEM270
	Gain	74,375,400	74,730,726	355,326 bp		CLIP2^c^; GTF2IRD1; GTF2I^b^
Atyp Dup3	Gain	73,528,186	74,662,079	1,133,893 bp	SNP Array 6.0 (Affymetrix)	FZD9^d^; BAZ1B^d^; BCL7B; TBL2; MLXIPL; VPS37D; DNAJC30; BUD23; STX1A; MIR4284; ABHD11-AS1; ABHD11; CLDN3; CLDN4; METTL27; TMEM270; ELN: LIMK1; EIF4H; MIR590; LAT2; RFC2; CLIP2; GTF2IRD1; GTF2I^c^

All coordinates are based on GRCh38/hg38 assembly.

^a^Genes that are partially deleted.

^b^A portion of this gene lies within a low copy repeat and therefore the exact deletion or duplication breakpoint cannot be determined by diagnostic microarray or real-time qPCR. All of the unique portion of the gene lies within the CNV.

^c^Genes that are partially duplicated.

^d^These genes were demonstrated to be duplicated using real-time qPCR and the Infinium HumanMethylation450k array analysis, although they were not included in the duplication interval on the original diagnostic microarray.

## Results

Participants’ age at assessment, sex, and CNV origin (inherited or de novo) are shown in Table 2A and demographic information for the comparison groups used for the methylation analyses is provided in Table 2B. The cognitive and behavioral, and medical phenotypic characteristics of each participant are summarized in Table 3.

**Table 2:** (A) Demographic information for participants and (B) demographic information for control participants for methylation analyses.

A			
Participant	Age at assessment (years)	Sex	Inheritance of CNV
Atyp Del1	5.13	F	De novo; dizygotic twin does not carry CNV
Atyp Del2	8.25	F	De novo
Atyp Del3	11.93	M	Inherited
Atyp Dup1	5.85	M	Inherited
Atyp Dup2	5.04	F	De novo
Atyp Dup3	4.12	F	De novo

B			
Group	Mean age at blood draw (years ± SD)	Range (years)	Sex
WBS	6.1 ± 1.58	2.8–8.5	14F, 6M
Dup7	7.8 ± 2.44	4.4–10.7	5F, 5M
TD	5.5 ± 1.43	2.4–7.4	12F, 3M

*SD* standard deviation, *TD* typically developing, *F* female, *M* male.

**Table 3:** Participants’ phenotypic relation to classic Williams-Beuren syndrome or 7q11.23 duplication syndrome.

Participant	Cognitive phenotype	Behavioral phenotype	Medical phenotype
Atyp Del1	Similar pattern of relative strengths and weaknesses^a^ Overall intellectual ability high for WBS but 1 SD below her TD twin	Similar levels of social pragmatic difficulties Similar levels of inattention and hyperactivity Sensitive to loud noises	WBS facies Hyperopia Chronic otitis media Ventricular septal defect Gastroesophageal reflux in infancy
Atyp Del2	Similar pattern of relative strengths and weaknesses Overall intellectual ability ~1.5 SD > WBS mean	Similar levels of social pragmatic difficulties Similar levels of inattention and hyperactivity Sensitive to loud noises	WBS facies Mild supravalvar aortic stenosis Multicystic dysplastic kidney Tremor
Atyp Del3	Different pattern of relative strengths and weaknesses Overall intellectual ability >2 SD above WBS mean	Considerably better social pragmatic skills Inattentive level in average range for TD children Considerably more hyperactive	Does not have WBS facies Microcephaly Difficulty gaining weight Short stature Gastroesophageal reflux Chronic constipation
Atyp Dup1	Speech-Sound Disorder that is not characteristic of Dup7 Developmental delay, most likely due to prematurity	Social pragmatic abilities in average range for TD children Inattentive and hyperactivity levels in average range for TD children	Does not have Dup7 facies Strabismus Joint laxity Hypospadias, cryptorchidism Developmental Coordination Disorder
Atyp Dup2	Speech-Sound Disorder that overlaps with what is typical for Dup7 Overall intellectual ability within typical range for Dup7	Similar levels of social pragmatic difficulties as for Dup7 Inattentive and hyperactivity levels in average range for TD children Social Phobia	Dup7 facies Macrocephaly Hypotonia Sleep disorder High pain tolerance Developmental Coordination Disorder
Atyp Dup3	Speech-Sound Disorder that is characteristic of Dup7 Overall intellectual ability > 2 SD above Dup7 mean	Social pragmatic abilities in average range for TD children Inattentive and hyperactivity levels in average range for TD children	Dup7 facies Hypotonia Chronic constipation

*SD* standard deviation, *TD* typically developing.

^a^Typically developing twin showed same pattern, so may be familial rather than related to WBS.

### Clinical findings

Atypical deletion individual 1 (Atyp Del1) is one of a dizygotic twin pair; her same-sex twin does not have a deletion at 7q11.23. Clinical assessment using the Differential Ability Scales-II (DAS-II)^30^ identified a similar pattern of relative strengths and weaknesses as is typical for classic WBS, in the context of a considerably higher level of overall ability. Verbal and nonverbal reasoning ability were within the average range for the general population, with spatial ability in the borderline range. A similar pattern of relative strengths and weaknesses was observed in the fraternal same-sex twin, suggesting that the relative weakness in spatial skills is likely familial. However, the unaffected twin’s overall intellectual ability (DAS-II^30^ General Conceptual Ability (GCA)) was 15 points higher.

Based on parental responses on the Social Responsiveness Scale-2 (SRS-2)^31^, Atyp Del1 was found to have social pragmatic abilities within the average range for children with WBS (considerably more limited than children her age in the general population). Parental rating on the Conners Early Childhood (EC)^32^ Inattention/Hyperactivity scale was within the average range for children with classic WBS (significantly more difficulty than children her age in the general population). The unaffected twin scored in the average range for children in the general population on both measures. Based on the Anxiety Disorders Interview Schedule for DSM-IV Parent (ADIS-P)^33^ interview, Atyp Del1 also had sensitivity to loud noises. Overall, this individual’s cognitive phenotype was similar to that of children with classic WBS, although her difficulty with spatial skills may be familial rather than deletion-related and her overall intellectual ability was higher than expected for classic WBS.

On physical examination, Atyp Del1 was found to have facial features characteristic of WBS. Review of medical records indicated hyperopia, a history of chronic otitis media, a ventricular septal defect, and gastroesophageal reflux as an infant.

Atypical deletion individual 2 (Atyp Del2) showed a similar pattern of relative strengths and weaknesses to those observed in the classic WBS comparison group; however, her spatial ability was higher than that of any child her age in the classic WBS group. Her overall intellectual ability (DAS-II^30^ GCA) was considerably higher than that of the classic WBS group of children her age. Her SRS-2^31^ T-scores indicated similar levels of pragmatic difficulty as children with classic WBS her age, and her Conners Comprehensive Behavior Rating Scales (CBRS)^34^ T-scores for the ADHD-Predominantly Inattentive and ADHD-Predominantly Hyperactive/Impulsive scales were within the average range for individuals with classic WBS her age. Based on the ADIS-P^31^ interview, she was sensitive to loud noises. Thus, her behavioural and cognitive phenotype was similar to that of same-aged children with classic WBS, although her overall intellectual ability and spatial ability were higher than expected for children with classic WBS.

Physical examination revealed facial features characteristic of WBS and a tremor. Review of medical records indicated that Atyp Del2 had mild supravalvar aortic stenosis and a multicystic dysplastic kidney.

Atypical deletion individual 3 (Atyp Del3) had a DAS-II^30^ GCA within the average range for children in the general population, despite a considerably lower socioeconomic status than most children in the WBS comparison group. He did not exhibit the characteristic pattern of relative strengths and weaknesses seen in classic WBS; instead, he showed a flat profile, with his spatial cluster standard score non-significantly higher than his verbal and nonverbal reasoning standard scores. Based on the SRS-2^31^, his social-pragmatic abilities were considerably stronger than those of children with classic WBS his age. Parental ratings on the Conners CBRS^34^ indicated that he had considerably more symptoms of ADHD-Predominantly Hyperactive/Impulsive but considerably fewer symptoms of ADHD-Predominantly Inattentive than is characteristic of children with classic WBS his age. This child did not show overlap with the common cognitive or behavioral features of the WBS comparison group.

On physical examination, Atyp Del3 had microcephaly and short stature. Review of medical records indicated difficulty gaining weight, gastroesophageal reflux, and chronic constipation. These characteristics are consistent with the WBS medical phenotype, although unlike most children with classic WBS, Atyp Del3 did not have any heart disease.

Atypical duplication individual 1 (Atyp Dup1) was born prematurely. He had developmental delay, most likely due to prematurity. His Speech Sound Disorder, which was not of the type associated with classic Dup7, also likely was due to prematurity. Unlike the classic Dup7 comparison group, this individual’s SRS-2^31^ Social Motivation T-score was in the average range for children in the general population, as were his social-pragmatic abilities overall. Based on the Conners EC^32^, his level of inattention/hyperactivity symptoms was average for the general population. He was classified as non-spectrum based on the Autism Diagnostic Observation Schedule-2 (ADOS-2) classification^35^ and clinical diagnosis. Other than developmental delay, his cognitive and behavioral phenotypes did not overlap with classic Dup7.

Physical examination revealed strabismus, joint laxity, and Developmental Coordination Disorder. He had mild cerebral palsy, likely due to prematurity. Review of medical records showed a history of hypospadias and cryptorchidism. He did not have facial features or other medical characteristics associated with classic Dup7.

Atypical duplication individual 2 (Atyp Dup2) performed in the average range for children her age with classic Dup7 on the DAS-II^30^ verbal, nonverbal reasoning, and spatial clusters and for overall intellectual ability. She also had a Speech Sound Disorder that overlapped with that associated with classic Dup7 and was diagnosed with Social Phobia based on ADIS-P interview^33^. Based on the SRS-2^31^, her level of social-pragmatic difficulties was typical for children with classic Dup7 her age. Her inattentive/hyperactive symptoms as measured by the Conners EC^30^ were at the level expected for children her age in the general population. She was classified as non-spectrum based on the ADOS-2 classification^35^ and clinical diagnosis.

Physical examination revealed facial features characteristic of children with Dup7, macrocephaly, hypotonia, and Developmental Coordination Disorder. Her parents reported that she had a high pain tolerance and a sleep disorder.

Atypical duplication 3 (Atyp Dup3) had a DAS-II^30^ GCA in the above average range for the general population. Her standard scores (SSs) for all three of the DAS-II core clusters were in the average to above average range for the general population. She was diagnosed with a Speech Sound Disorder pattern characteristic of children with classic Dup7. Her social-pragmatic skills as measured by the SRS-2^31^ and her inattention/hyperactivity symptoms as measured by the Conners EC^32^ were in the average range for the general population. She was classified as non-spectrum based on the ADOS-2 classification^35^ and clinical diagnosis.

Physical examination revealed facial features typical of classic Dup7. She also had hypotonia. Review of medical records indicated that she had chronic constipation.

### Molecular analyses

**Fig. 1:**
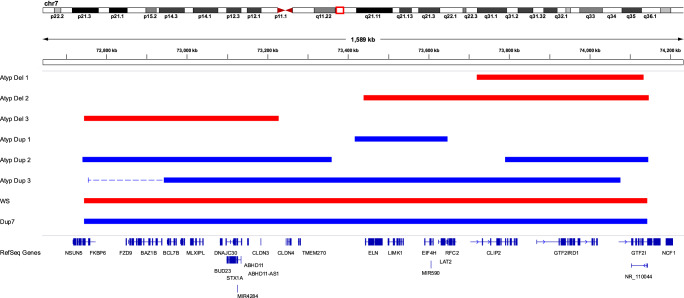
Overview of atypical 7q11.23 CNV genotypes. Shown is an overview of the atypical deletion and duplication participants identified in our study. Red bars indicate deletions and blue indicate duplications. 7q11.23 CNVs were identified by chromosomal microarray analysis (CMA) and confirmed by qPCR, using a standard panel of genes across the region. The proximal breakpoint of atypical duplication 3 (Atyp Dup3) was found to be more proximal by qPCR than what was reported by CMA. The region shown as duplicated by qPCR is marked by a dashed line. All other breakpoints were concordant between CMA and qPCR. Included is a representative WBS deletion and the reciprocal duplication characteristic of Dup7.

Previous analyses of DNA methylation in cohorts of children with classic WBS, children with classic Dup7, and typically developing (TD) controls identified genome-wide dose-dependent changes to DNA methylation^19^. Each individual analyzed in our previous study harbored a typical 7q11.23 deletion or duplication (concordant breakpoints) and classic phenotype; therefore, our previous study could not elucidate which gene(s) within this region could be contributing to the methylation changes we observed. To better understand the molecular mechanism accounting for the methylation changes we detected, we recruited several individuals with rare, atypical deletions or duplications of the 7q11.23 region (Table 1), generated DNA methylation profiles, and compared them to those reported previously^19^. An overview of the CNVs analyzed in this study is shown in Fig. 1. This proof-of-principle study was employed to determine if a small number of rare deletions and duplications could aid in elucidating the genes contributing to aberrant DNA methylation in disorders of 7q11.23 CNV.

The distal breakpoint of both the classic WBS deletion and the Dup7 duplication lie within a flanking low copy repeat, making it hard to determine whether CNV of this region includes the full GTF2I gene. The distal breakpoints of three of the atypical CNVs also lie within the low copy repeats making accurate determination of the exact boundary impossible by microarray or qPCR. To help determine whether *GTF2I* was impacted in these participants, expression analysis of *GTF2I* was carried out by real-time qPCR. Participants with deletion of *GTF2I* (Atyp Del1 and Atyp Del2) showed transcript levels that were consistent with those seen in individuals with WBS. Participants Atyp Del3 and Atyp Dup1 who had two genomic copies of *GTF2I* showed corresponding transcript levels similar to the control group with no 7q11.23 CNV (Supplementary Table 2). Participant Atyp Dup3, with only a partial duplication of *GTF2I* encompassing the first few exons, also showed *GTF2I* transcript levels similar to the control group when primers within the non-duplicated region of the gene were used.

To determine whether expression of genes within the common 7q11.23 CNV that have the potential to affect DNA methylation (*BAZ1B*, *BCL7B*, or *BUD23*) were altered by nearby genomic rearrangements, transcript levels were assessed in the three participants with CNVs that did not change the copy number of these genes (Atyp Del1, Atyp Del2, and Atyp Dup1). All three participants had transcript levels that were similar to those from control participants, and distinctly different from individuals with WBS or Dup7.

Overall, expression analysis of *GTF2I* as well as genes within the common 7q11.23 CNV that could affect DNA methylation (*BAZ1B*, *BCL7B*, *BUD23*), support the findings from genomic copy number analysis.

### DNA methylation analyses

**Fig. 2:**
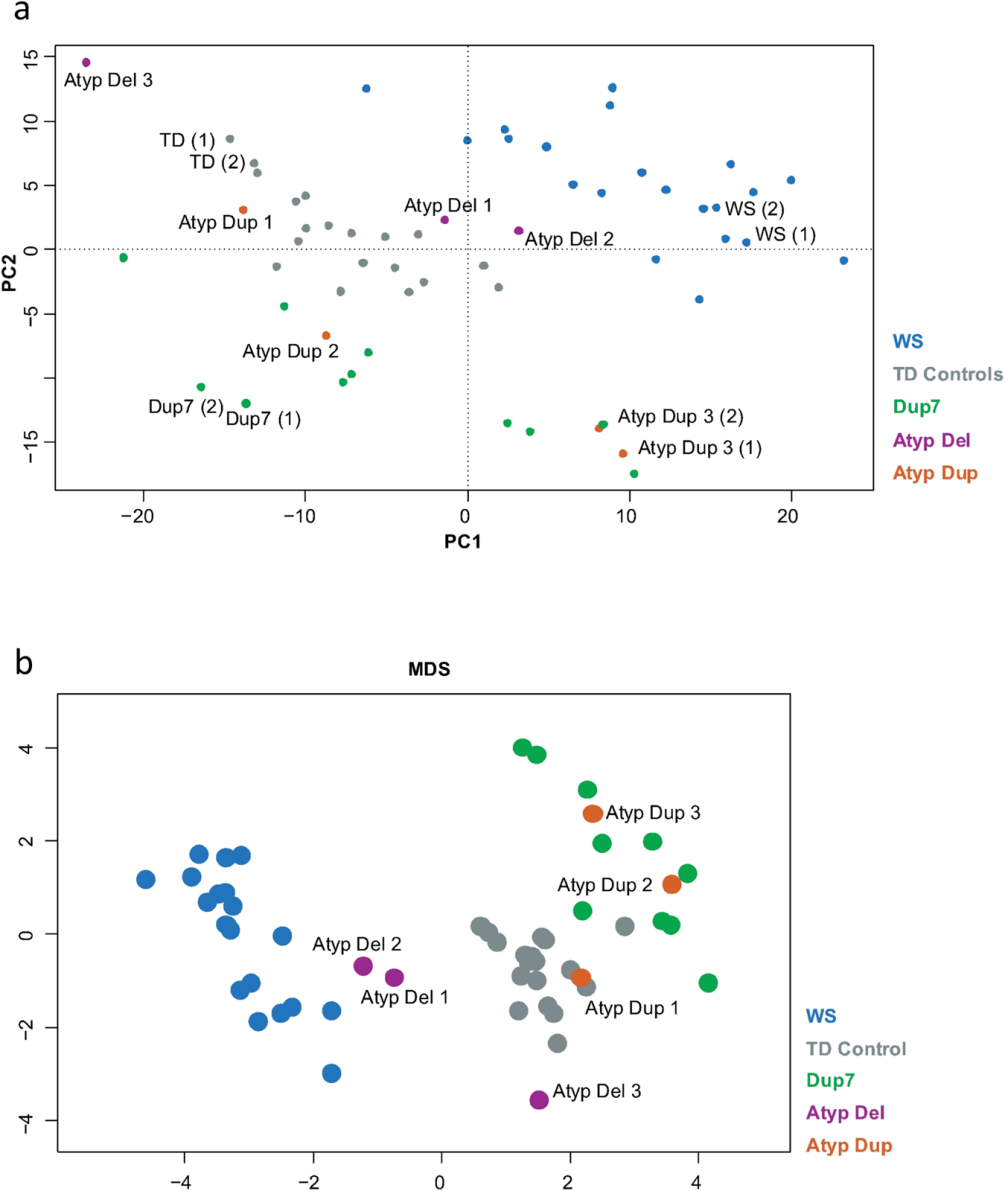
DNA methylation profiles. **a** PCA of methylation profiles. Methylation profiles from the six participants with atypical 7q11.23 deletions (purple) or duplications (orange) are shown alongside those from children with classic WBS (blue), children with classic Dup7 (green) and typically developing (TD) children (gray). Technical replicates (two from each group) are noted with their relative batch in parentheses. **b** Multi-dimensional scaling (MDS) plot of the top 1000 most variable positions across all individuals. Three distinct clusters are resolved representing children with WBS (blue), children with Dup7 (green), and TD children (gray). Participants with atypical CNVs differentially cluster with the classic WBS, classic Dup7 and TD groups in a genotype-dependent manner.

Principal component analysis (PCA) of DNA methylation data from all participants, including technical replicates, indicated that each atypical rearrangement exhibited a different DNA methylation profile, suggesting that the differing genotypes are driving changes to DNA methylation profiles (Fig. 2a). Analysis of all samples, including technical replicates, confirmed that the results were not confounded by batch effects (Fig. 2a). Re-analyses of previously generated data^19^ in conjunction with new data from participants with atypical 7q11.23 CNV again resulted in the clustering of the WBS group separate from the Dup7 group, which were separate from the TD controls (Fig. 2a). Notably, the DNA methylation patterns of several atypical participants appeared to cluster with their respective syndromic cohort. Atyp Dup2 and Atyp Dup3 (both technical replicates 1 and 2) clustered closely with the classic Dup7 cohort, whilst both Atyp Del1 and Atyp Del2 fell between TD controls and the classic WBS cluster. Atyp Dup1 clustered closely with TD controls, and Atyp Del3 was closer to the TD controls than the WBS cluster but did appear noticeably different from the TD group (Fig. 2a).

Atyp Del2 and Atyp Del3 did not clearly cluster with either TD controls or the WBS cluster, suggesting that the shared deletion of telomeric genes at 7q11.23 contributes to changes in DNA methylation but may not be sufficient to replicate the entire methylation profile. To better understand the contribution of these genes to larger gains and losses of DNA methylation, we assessed the top 1000 most variable positions using multi-dimensional scaling (MDS; Fig. 2b). Technical replicates were first removed, and the entire batch reanalyzed; this was to ensure that the results of this small study were not skewed by the inclusion of similar samples. Analysis of these positions again replicated the distinct clustering of WBS, Dup7, and TD control cohorts, validating this approach. Atypical participants now clustered in a much clearer genotype-dependent manner; Atyp Dup1 was indistinguishable from controls, Atyp Dup2 and Atyp Dup3 clustered with the classic Dup7 group, Atyp Del3 clustered closer to the TD controls, and Atyp Del1 and Atyp Del2 again clustered between WBS group and TD controls but closer to the classic WBS group (Fig. 2b).

**Fig. 3:**
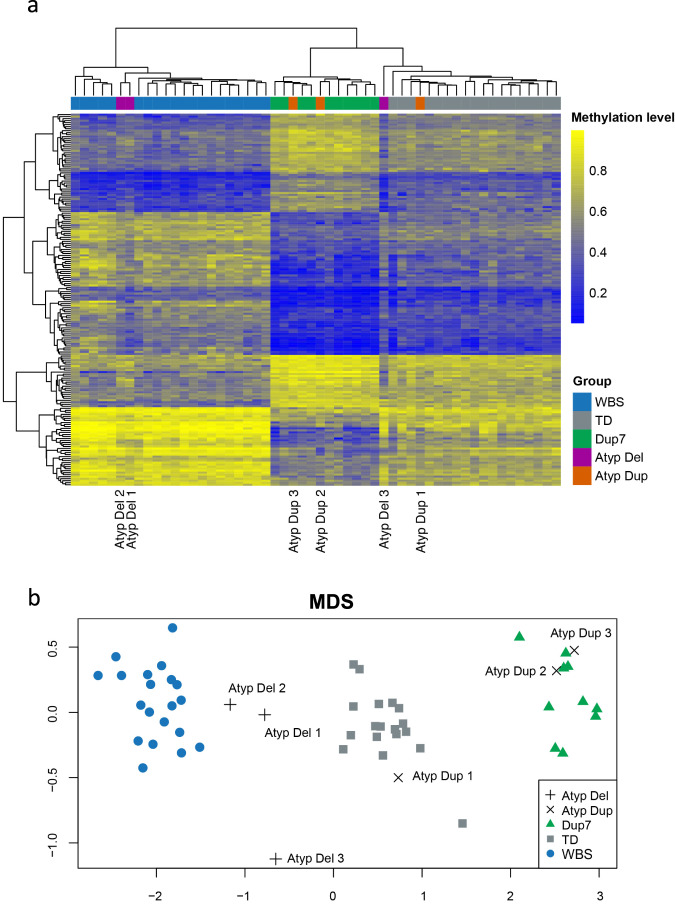
Heatmap with hierarchical clustering and MDS analyses of a subset of 185 distinguishing CpG sites. **a** Heatmap with hierarchical clustering across 185 CpG sites found to be significantly differentially methylated in both the WBS to TD and Dup7 to TD comparisons and (**b**) MDS plot of the same data replicates the profile generated by hierarchical clustering.

Previous analyses of DNA methylation identified a dose-dependent profile consisting of a small number of CpG sites that could distinguish individuals with WBS, TD controls, and individuals with Dup7^19^. This strategy was re-employed in this study to better understand how the atypical participants grouped across highly distinguishing CpG sites. Analysis of 185 CpG sites that are significantly differentially methylated in both WBS and Dup7 cohorts again clearly distinguished each cohort (Fig. 3). Hierarchical clustering grouped both Atyp Del1 and Atyp Del2 with the WBS cohort, whilst Atyp Dup2 and Atyp Dup3 clustered with the Dup7 cohort (Fig. 3a). Atyp Del3 and Atyp Dup1 both clustered with TD controls; however, Atyp Del3 is noted as having a methylation profile distinct from the other groups (Fig. 3a).

Across several clusters of CpG sites in the heatmap, Atyp Del3 demonstrated a methylation pattern similar to the WBS group, suggesting that although deletion of the centromeric end is not sufficient to replicate the global methylation pattern observed in WBS participants, there is likely some contribution of the centromeric genes to aberrant DNA methylation (Fig. 3a). Without a second atypical participant sharing the same or similar deletion as Atyp Del3, it is difficult to draw firm conclusions from the methylation profile of this single participant. A MDS plot of the same data replicated these findings and produced an overall profile similar to that observed across the top 1000 most variable positions (Fig. 3b).

The methylation profile across the top 500 most differentially methylated (DM) CpG sites in each cohort were evaluated. Both absolute differential methylation (topmost differentially methylated sites) and the top 250 hyper- and 250 hypomethylated CpG sites were assessed. Hierarchical clustering produced variable results within the WBS to TD comparison, with clustering across the top 500 most differentially methylated CpGs resulting in all atypical deletion participants clustering with TD controls (Supplementary Fig. 1A); however, the associated MDS plot again demonstrated Atyp Del1 and Atyp Del2 clustering between WBS and TD groups (Supplementary Fig. 1B). Analysis of the top 250 most hyper- and 250 most hypomethylated CpGs in the WBS to TD comparison clearly clustered Atyp Del1 and Atyp Del2 with the WBS cohort and Atyp Del3 with TD controls, consistent with all previous analyses (Supplementary Fig. 1C, D). This apparent discrepancy may be due to technical differences between hierarchical clustering and MDS analyses or may suggest a specific influence of the genes encompassed within Atyp Del1 and Atyp Del2 to the more numerous hypomethylated CpGs captured within the top 250 hyper- and 250 hypomethylated CpGs.

The methylation profiles generated from the Dup7 to TD comparison again consistently clustered Atyp Dup2 and Atyp Dup3 with the Dup7 cohort, and Atyp Dup1 with TD controls, regardless of which CpG sites were assessed, consistent with all previous approaches to analysis (Supplementary Fig. 2). These analyses replicate the results observed across the top 1000 most variable positions and the top discriminating CpG sites, supporting the conclusion that atypical deletion or duplication of this region can produce distinct methylation profiles.

Validation of methylation levels was performed across selected CpG sites from the two top-most DM genes from our initial study (*ANKRD30B* and *RFPL2*)^19^, using targeted pyrosequencing. The correlations between methylation levels detected by microarray and by pyrosequencing were very high, and differences between atypical participants could be resolved (Supplementary Fig. 3). Overall, the pyrosequencing data supported the global DNA methylation patterns for each of the participants with atypical 7q11.23 CNV.

In total, three different methods of assessing DNA methylation have demonstrated that the participants with atypical 7q11.23 deletions and duplications have methylation profiles that differ by genotype. All three methods suggest that genes at the telomeric end of 7q11.23 contribute to the larger changes in DNA methylation observed in our cohort, although a smaller contribution of genes toward the centromeric end is also likely.

## Discussion

The identification and subsequent analysis of individuals with atypical rearrangements of 7q11.23 has been important to our understanding and evaluation of genotype-phenotype correlations in WBS and Dup7. Such rearrangements are rare, and their clinical significance is challenging to interpret. Larger, atypical rearrangements that extend distally past the typical breakpoint constitute less than 5% of the population of individuals with clinically reported CNV at 7q11.23^8,9^. The prevalence of small, atypical deletions or duplications has not been established. We have previously shown that deletion or duplication of 7q11.23 leads to dose-dependent genome-wide changes to DNA methylation in peripheral blood-derived DNA from children with classic rearrangements of this locus^19^. Numerous genes within the common 7q11.23 CNV have been associated with epigenetic complexes; therefore, it was difficult to predict which gene or combination of genes contributed to the DNA methylation profiles observed in that study.

The present study was designed as a proof-of-principle, whereby a small number of individuals with rare, atypical deletions or duplications at 7q11.23 were profiled to determine if atypical genotypes may help unravel the molecular underpinnings of aberrant DNA methylation at this locus. We have demonstrated that this is indeed possible, and although our cohort is currently small, several important conclusions can be drawn from these results.

Each atypical deletion or duplication carried a unique genotype, with variably deleted genes at the centromeric and telomeric ends, and variable contribution of genes previously associated with epigenetic mechanisms. Atyp Dup1 consistently clustered closely with controls, suggesting that CNV for the genes encompassed by this duplication (*ELN* to *LAT2;* Fig. 1) does not contribute significantly to altered DNA methylation across the top differentially methylated CpGs, at least with respect to copy number gain. From a molecular standpoint, this was expected, as none of the genes within this region has been associated with epigenetic mechanisms. One microRNA (MIR590) is found at this locus, and some microRNAs have been shown to play a role in regulating DNA methylation^36^. MIR590 has been implicated in numerous types of cancer, either as an oncogene or as a tumor suppressor depending on its transcriptional target^37^. Our data suggest that this microRNA is not a major contributor to the methylation profiles detected in individuals with WBS or Dup7, a conclusion that is supported by the reciprocal duplication seen in Atyp Dup2, who has a complex rearrangement that duplicates all genes within the common 7q11.23 CNV except those duplicated in Atyp Dup1, and who consistently clusters with the classic Dup7 cohort. From a clinical standpoint, Atyp Dup1 and Atyp Dup2 have very different presentations. Whereas Atyp Dup1 has minimal overlap with classic Dup7 phenotypes, Atyp Dup2 shares similar cognitive, behavioral, and medical phenotypes with a comparative cohort of similarly aged individuals with classic Dup 7 (Table 3). Our data mirror the clinical phenotypes of Atyp Dup1 and Atyp Dup2, suggesting that DNA methylation may be an important contributor to the pathophysiology of this disorder.

Atypical deletions 1–3 are particularly informative as they show differing deletions of the centromeric (Atyp Del3) and telomeric (Atyp Del1 and Atyp Del2) genes associated with epigenetic mechanisms (Fig. 1). Genome-wide analysis of DNA methylation profiles across Atyp Del1 and Atyp Del2 suggest that CNV for genes at the telomeric end of 7q11.23, namely *GTF2I* and *GTF2IRD1*, likely strongly contributes to altered DNA methylation, particularly across the large changes in DNA methylation observed in our dataset. Loss of these genes results in global methylation profiles that cluster between WBS and TD controls across multiple analyses. This is supported by analyses of Atyp Dup2 and Atyp Dup3 whose DNA methylation profiles cluster closely with the classic Dup7 cohort.

The only genes commonly rearranged across all four individuals are *CLIP2* and *GTF2IRD1*, suggesting that this may be the minimal region that drives large changes in DNA methylation. CLIP2 is a component of the microtubule network and is highly expressed in the brain, and there is currently no evidence to support a role for this protein in epigenetic pathways or mechanisms^38,39^. The involvement of other genes from the 7q11.23 region linked to DNA methylation is also unlikely, given that their genomic copy number does not correlate with DNA methylation profile (Table 4) and their expression is not altered by non-overlapping CNVs (Supplementary Table 3).

**Table 4:** Summary of functional copy number and DNA methylation profile for participants with atypical 7q11.23 Copy Number Variants (CNVs).

Participant	Gene					DNA methylation profile^b^
	BAZ1B	BCL7B	BUD23	GTF2IRD1^a^	GTF2I	
Atyp Del1	2 copies	2 copies	2 copies	1 copy	1 copy	WBS
Atyp Del2	2 copies	2 copies	2 copies	1 copy	1 copy	WBS
Atyp Del3	1 copy	1 copy	1 copy	2 copies	2 copies	TD
Atyp Dup1	2 copies	2 copies	2 copies	2 copies	2 copies	TD
Atyp Dup2	3 copies	3 copies	3 copies	3 copies	3 copies	Dup7
Atyp Dup3	3 copies	3 copies	3 copies	3 copies	2 copies	Dup7

^a^*GTF2IRD1* expression could not be assayed due to very low levels.

^b^DNA methylation profile refers to the group profile that each participant most closely clustered with after multi-dimensional scaling (Fig. 2b).

Atyp Dup2 and Atyp Dup3 both cluster with the classic Dup7 cohort but the first participant has a rearrangement encompassing the entire *GTF2I* gene, whilst the second does not. The exact breakpoint of Atyp Dup3 is still undefined, but microarray analysis shows that it extends only past the first non-coding exon of *GTF2I*, and this is supported by real-time qPCR analysis that demonstrates normal copy number of *GTF2I* exon 4. Real-time QPCR expression analysis using an amplicon spanning exons 10 to 12 showed expression equivalent to two copies of *GTF2I* (Supplementary Table 2). Based on these assays it is highly unlikely that the level of TFII-I protein is altered by the CNV in this individual. The GTF2I proteins, TFII-I and TFII-IRD1, are members of the TFII-I family of transcription factors that arose from the same common ancestor and share sequence similarity^40^. Several studies suggest that these proteins share overlapping functions and bind to similar targets^41,42^, so it is possible that both contribute to the altered DNA methylation associated with CNV at 7q11.23.

Although this study was designed from a molecular perspective, it is interesting to note that each participant had a different clinical phenotype that somewhat mirrors their respective methylation profile. Atyp Del1 and Atyp Del2 had clinical phenotypes that recapitulated many features of the classic WBS cohort, albeit with higher overall intellectual ability. Atyp Del3 did not have the characteristic cognitive, behavioral, or facial features of classic WBS, although he did share several characteristic medical features. This individual had a methylation profile that was unlike any of the other individuals in the study, suggesting there may be an alternative molecular contribution to his methylation profile.

Atyp Dup2 and Atyp Dup3 shared the Speech-Sound Disorder characteristics, hypotonia, and facial features that are characteristic of classic Dup7, and both individuals had methylation profiles that clearly clustered with the Dup7 cohort, in line with these important clinical features. However, overall, there was more phenotypic overlap with classic Dup7 for Atyp Dup2 than for Atyp Dup3. Atyp Dup2 had macrocephaly, a high pain tolerance, Social Phobia, Developmental Coordination Disorder, and social-pragmatic difficulties in the range typical for individuals with Dup7, none of which was present in Atyp Dup3. Atyp Dup1 showed no significant overlap with Dup7 clinically; this individual’s methylation profile was indistinguishable from controls. The fact that the results of these analyses mirror each individual’s clinical presentation poses an interesting question: can methylation profiles be used to predict the clinical profile of individuals with copy number changes at 7q11.23?

Atypical rearrangements at 7q11.23 are typically only brought to attention when a clinically affected individual undergoes diagnostic testing via chromosomal microarray. The clinical significance of such rearrangements is uncertain given their rarity and lack of information regarding the pathogenicity of deletion or duplication of isolated genes at 7q11.23. Having an additional genetic test that would help to understand the possible pathogenicity of such changes would be very valuable from a clinical perspective. DNA methylation has recently been used in genomic diagnostic testing for a similar purpose, particularly to help elucidate the pathogenicity of variants of uncertain significance in genes associated with chromatin remodeling complexes^43–45^. Our data support the hypothesis that in the future a similar strategy could be employed for variants within 7q11.23. Additional work will be required to refine and validate a robust methylation signature that may further aid in teasing out the molecular contribution of 7q11.23 to DNA methylation.

In conclusion, we have shown that atypical deletions and duplications at 7q11.23 result in differing DNA methylation profiles. Comparative analysis of these profiles to those of individuals with classic WBS, classic Dup7, or TD controls suggests that the telomeric genes *GTF2I* and *GTF2IRD1* play a significant role in DNA methylation processes. We have also provided evidence to support the potential use of DNA methylation in elucidating the clinical significance of rearrangements of 7q11.23.

## Methods

### Research participants

All molecular genetic procedures were approved by the Research Ethics Board of the University of Toronto, all procedures involving medical data were approved by the Institutional Review Board of the University of Nevada, Reno, and all procedures involving psychological data were approved by the Institutional Review Board of the University of Louisville. Written informed consent was obtained from parents or guardians of all participants, and oral or written assent was obtained from the participants. 7q11.23 deletion or duplication size and gene content were determined by CNV microarray analysis (detailed in Table 1) and confirmed by real-time PCR analysis. An overview of the individual CNVs is shown in Fig. 1. Participants’ age at assessment, sex, and CNV origin (inherited or de novo) are shown in Table 2A. Demographic information for the comparison groups used for the methylation analyses is provided in Table 2B.

Clinical and psychological assessment results for each participant were compared to those from large comparison groups of children with classic WBS or classic Dup7 from the University of Louisville cohorts, including published reports for children with WBS^46^ and Dup7^47^.

### Psychological, behavioral, and medical characteristics

Intellectual ability and patterns of relative strength and weakness were measured using the Differential Ability Scales-II (DAS-II)^30^, which provides standard scores (SSs, mean = 100, SD = 15 for the general population) for verbal ability, nonverbal reasoning ability, and spatial ability as well as overall intellectual ability (General Conceptual Ability (GCA); similar to IQ). The Social Responsiveness Scale-2 (SRS-2)^31^, which provides T-scores (mean = 50, SD = 10 for the general population) was used to measure social-pragmatic abilities. The Conners Early Childhood (EC; ages 2–5 years)^32^ and Conners Comprehensive Behavior Rating Scales (CBRS; ages 6–18 years)^34^, which provide T-scores, were used to measure attention problems. The SRS-2, EC, and CBRS were completed by participants’ mothers. Social Phobia was diagnosed and sensitivity to loud noises was identified based on the Anxiety Disorders Interview Schedule for DSM-IV Parent interview schedule (ADIS-P)^33^ and autism spectrum disorder (ASD) was assessed using the Autism Diagnostic Observation Schedule-2 (ADOS-2)^35^ along with clinical judgment. Each participant’s performance on these measures was compared to that of a large group of individuals of similar age who had classic WBS (for the children with atypical deletions) or classic Dup7 (for individuals with atypical duplications). Participants with Dup7 also were evaluated for Speech Sound Disorder by a licensed speech-language pathologist who had considerable experience with children with Dup7. Speech Sound Disorder is not a major phenotypic characteristic of WBS, so these individuals were not assessed for that. The cognitive and behavioral phenotypic characteristics of each participant are summarized in Table 3.

Facial features characteristic of WBS or Dup7 were identified and microcephaly, macrocephaly, hypotonia, and Developmental Coordination Disorder were diagnosed based on clinical genetics exam. Cardiac disease and other medical problems were identified from review of medical records. The medical phenotypical characteristics of each participant also are reported in Table 3.

### Infinium HumanMethylation450K array analyses

DNA was extracted from 200 μl of buffy coat cells using QIAamp DNA Blood Mini Kit (Qiagen). In all, 1 μg of DNA was bisulfite converted using the Epitect Bisulfite Kit (Qiagen) following manufacturer’s recommendations. In total, 500 ng of bisulfite converted DNA was hybridized to the Infinium HumanMethylation450k microarray and the remaining 500 ng of DNA was reserved for targeted pyrosequencing analysis. Pyrosequencing was performed as previously described^16^.

The goal of this experiment was to establish methylation profiles from individuals carrying atypical deletions or duplications of 7q11.23 and to compare those profiles to those previously established from individuals with classic deletion or duplication of this region^19^. To enable this comparison and control for batch effects between experiments and between microarrays, three sets of technical replicates were included: one typically developing control, one participant with an atypical duplication (Atypical duplication individual 3: Atyp Dup3), and one participant with WBS. Each sample was previously included in our prior study^19^ and served as controls for batch effects between experiments. Aliquots from the original DNA samples were bisulfite converted alongside samples in the current study to minimize any technical variation and hybridized to the current batch of microarrays. All data, including those samples previously assessed^19^, were re-analyzed from the raw IDAT files and processed as a new experiment.

Raw image files were collected from the current dataset that includes the analyses of atypical deletion and duplication participants, and from the previously published dataset of WBS, Dup7 and TD controls^19^. All raw image data files were processed together using the R package minfi (R version 3.1.3)^48^. Various normalization protocols were tested for their ability to minimize batch effect and smooth methylation signals between and within batches, and correlation between technical replicates was measured. Illumina normalization, as employed using the R package minfi^48^, demonstrated the highest correlation between microarray and pyrosequencing methylation levels and therefore was implemented as the normalization method within this experiment.

Methylation data were first converted to M-values by logit transformation of beta-values as this method is favored for performing statistical analyses of methylation data^49^. Data were filtered to remove the following CpG sites: polymorphic sites with a minor allele frequency (MAF) of 0.05, CpG sites that were previously established as cross-reactive, probes that failed in at least one sample, and CpG sites on the X and Y chromosomes. This filtering step was performed using the “rmSNPandCH” function in the R package DMRcate (version 1.14.0)^50^. To account for differences in blood cell populations, the cellular composition of each sample (including those previously reported) was generated using the “estimateCellCounts” function within the R package minfi^48^.

The 1000 most variable positions were captured within the minifi pipeline using the function mdsPlot^36^. This functionality identifies the positions with the most variability in DNA methylation when calculating the distance between samples.

To control for sample mix-up, CNV was estimated using the R package conumee v1.4.2^51^. The genomic location of genes previously associated with epigenetic mechanisms was used to describe regions in finer detail, and highly polymorphic regions were excluded as per standard conumee processing. CNVs were identified by comparing each case to the TD control methylation signal. The results of this analysis are shown in Supplementary Fig. 4. These data support the original chromosomal microarray analysis data and thus confirmed sample identity within this experiment.

Differentially methylated CpGs were identified using the R package Limma (version 3.34.9)^52^. Estimated blood cell counts, the sex and age of participants were included as covariants in the linear model to control for biological sources of variation.

Differentially methylated CpGs were identified by contrasting WBS to TD control cohorts, Dup7 to TD control cohorts, and WBS to Dup7 cohorts, as previously described^19^, with Benjamini-Hochberg correction applied. The data were filtered for statistically significant CpG sites (*P* < 0.05). The average methylation level of each CpG site was calculated across each cohort and then the degree of methylation difference relative to the TD control cohort was determined by subtracting the average methylation level of the TD group from either the WBS or Dup7 group. The degree of differential methylation (delta beta) was further filtered to those sites with at least a 10% change in DNA methylation. All further analyses started from statistically significant CpG sites that showed at least a 10% change in methylation between each cohort.

Two strategies for evaluating methylation profiles were established. First, CpG sites that were statistically significant in both the WBS to TD and Dup7 to TD comparisons were identified. This strategy was utilized in our previous work to separate the different cohorts based on methylation profile and to demonstrate the symmetrical nature of these profiles across WBS and Dup7 participants^19^. Second, the top 500 CpGs showing the greatest absolute change in methylation (delta beta) and the top 250 hyper- and top 250 hypomethylated CpG sites were captured for each of the following comparisons: WBS to TD controls and Dup7 to TD controls. The data were visualized using a combination of heatmaps with hierarchical clustering (R package pheatmap, version 1.0.12)^53^ and multidimensional scaling plots (MDS). Both methods employed euclidean distance metrics.

### Expression analysis

RNA was extracted from whole blood collected in Tempus Blood Collection tubes (Life Technologies) using QiaAmp RNA blood mini kit (Qiagen), following the manufacturers protocol. 500 ng of RNA was converted to cDNA using Superscript III Reverse Transcriptase (Life Technologies), with the addition of a final RNase H (Life Technologies) treatment. In preparation for real-time PCR, cDNA samples were diluted 1 in 100 in nuclease free water. Real-time PCR was performed using Power SYBR Green PCR master mix (Life Technologies) and assessed using the ViiA7 detection system (Life Technologies). Standard curves were generated for each primer set and detected values were normalized to the housekeeping genes HMBS, HPRT1 and TBP. Real-time PCR assay primer sequences are shown in Supplementary Table 1. Genes included in the analysis were BAZ1B, BCL7B, BUD23 and GTF2I. High-quality RNA samples were available for all participants except for Atyp Dup2.

### Reporting summary

Further information on research design is available in the Nature Research Reporting Summary linked to this article.

### Supplementary information

Supplementary Materials

Reporting Summary

## Data Availability

The datasets used and/or analyzed during the current study are available from the corresponding author on reasonable request. Raw and normalized Illumina 450K methylation data for previously published comparison groups used for methylation analyses (Table 2B) can be accessed through the Gene Expression Omnibus GEO: GSE66552.
