# Supplementary material for: DNA methylation profiles in individuals with rare, atypical 7q11.23 CNVs correlate with GTF2I and GTF2IRD1 copy number
Source: NPJ Genom Med. 2023 Sep 14;8:25. doi: 10.1038/s41525-023-00368-7 (PMC10502022; doi:10.1038/s41525-023-00368-7)
Supplement: Supplementary file 1 — Supplementary Materials [file 41525_2023_368_MOESM1_ESM.pdf]

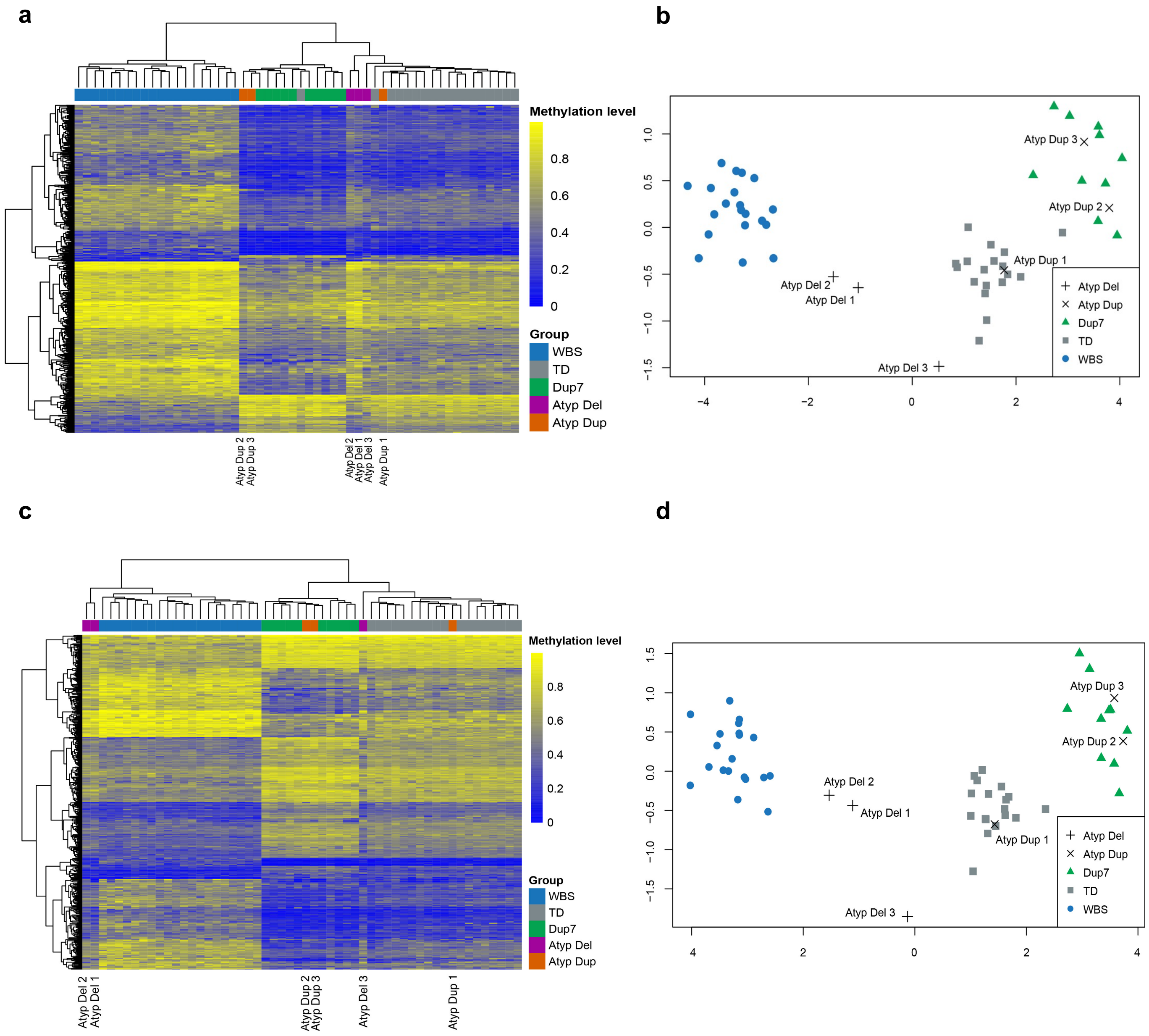

Supplementary Figure 1. Analysis of the top 500 most differentially methylated CpGs in the WBS to TD comparison. (a) Heatmap with hierarchical clustering across the top 500 most differentially methylated CpGs (absolute methylation difference) from the comparison between WBS and TD controls, and **(b)** MDS plot depicting spatial clustering across the same 500 CpGs. **(c)** Heatmap with hierarchical clustering across the top 250 most hypermethylated and 250 most hypomethylated CpGs from the WBS to TD comparison, and **(d)** MDS plot depicting the spatial clustering of each sample across the same hyper- and hypomethylated CpGs.

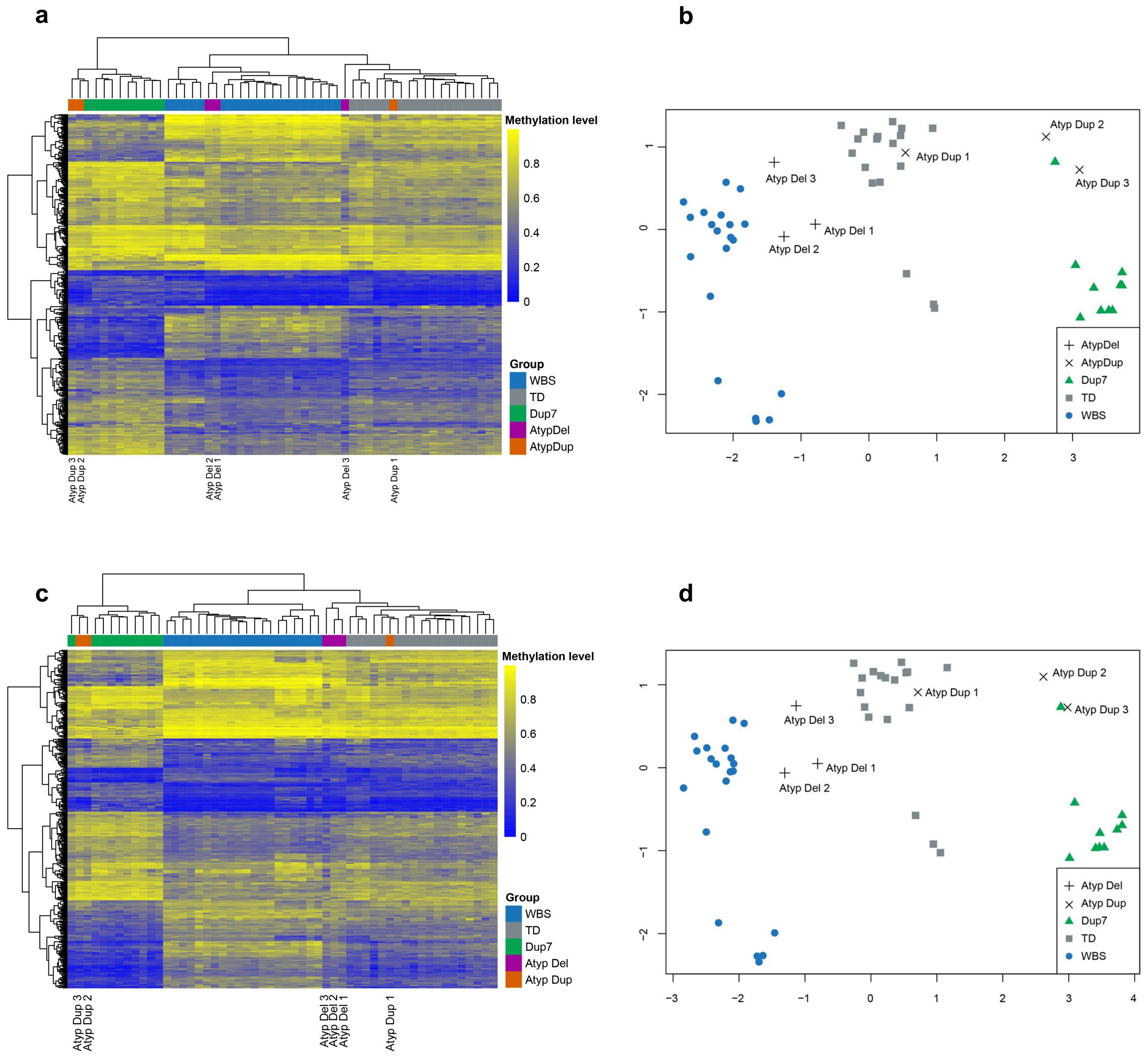

Supplementary Figure 2. Analysis of the top 500 most differentially methylated CpGs in the Dup7 to TD comparison. (a) Heatmap with hierarchical clustering across the top 500 most differentially methylated CpGs (absolute methylation difference), and **(b)** MDS plot showing the spatial clustering of participants across the same 500 CpGs. (**c**) Heatmap with hierarchical clustering across the top 250 most hyper- and 250 most hypomethylated CpGs, and **(d)** MDS plot depicting the spatial clustering of participants across the same hyper-and hypomethylated CpGs.

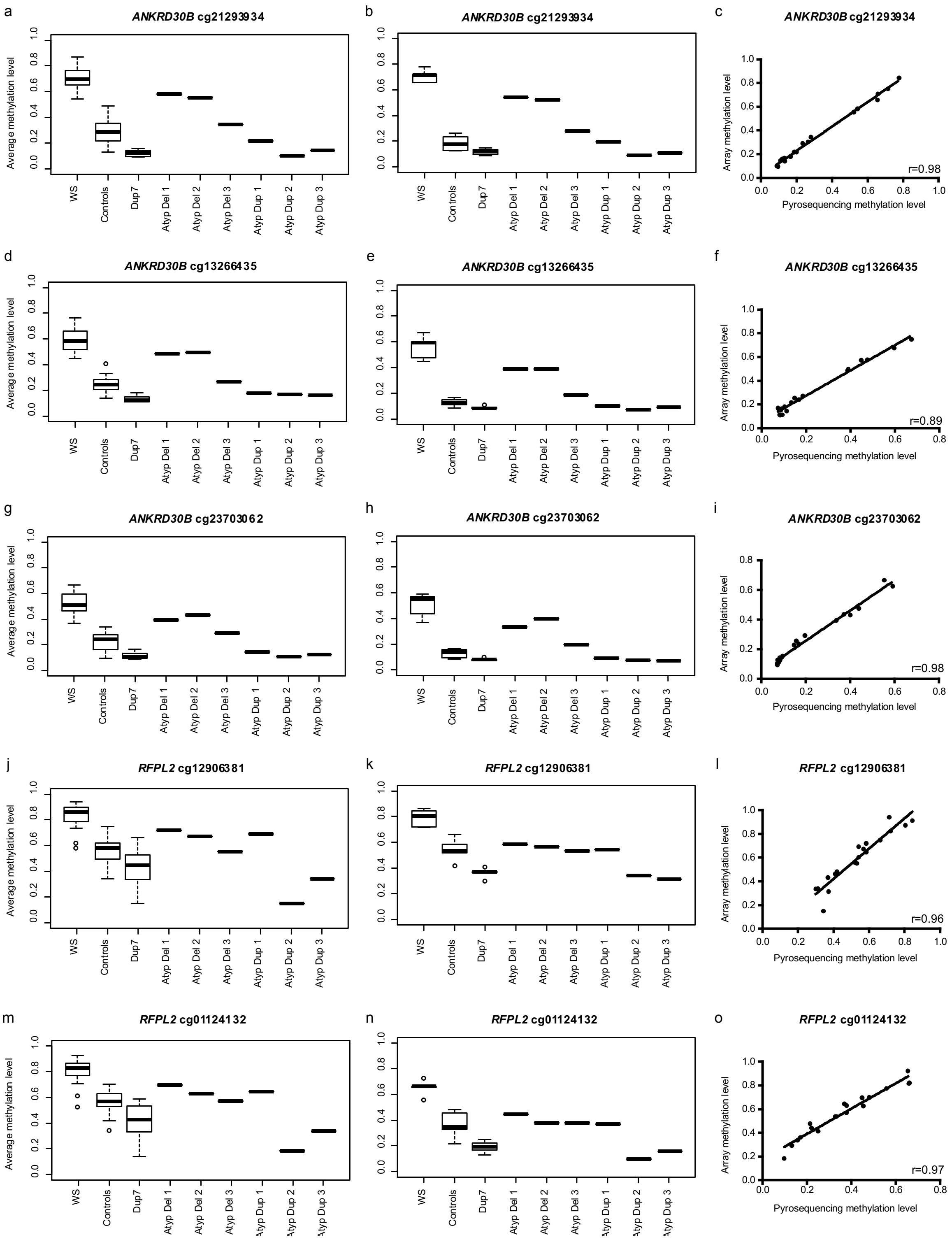

a
ANKRD30B cg21293934
Average methylation level
WS
Controls
Dup7
Atyp Del 1
Atyp Del 2
Atyp Del 3
Atyp Dup 1
Atyp Dup 2
Atyp Dup 3
b
ANKRD30B cg21293934
c
ANKRD30B cg21293934
Array methylation level
Pyrosequencing methylation level
r=0.98
d
ANKRD30B cg13266435
e
ANKRD30B cg13266435
f
ANKRD30B cg13266435
r=0.89
g
ANKRD30B cg23703062
h
ANKRD30B cg23703062
i
ANKRD30B cg23703062
r=0.98
j
RFPL2 cg12906381
k
RFPL2 cg12906381
l
RFPL2 cg12906381
r=0.96
m
RFPL2 cg01124132
n
RFPL2 cg01124132
o
RFPL2 cg01124132
r=0.97

Supplementary Figure 3. Pyrosequencing validation of CpG sites. (a) Boxplots depicting methylation levels across ANKRD30B cg21293934 as observed from microarray analysis. **(b)** Boxplots depicting the methylation levels from ANKRD30B cg21293934 obtained from secondary pyrosequencing analysis. **(c)** Spearman correlation analysis of methylation levels obtained from microarray and pyrosequencing. r represents Spearman correlation. **(d)** Methylation levels across ANKRD30B cg13266435 as identified from microarray analysis. **(e)** Pyrosequencing methylation levels across cg13266435. **(f)** Spearman correlation analysis of cg13266435 methylation levels identified by microarray and pyrosequencing. **(g)** Methylation levels across ANKRD30B cg23703062 identified from microarray analysis. **(h)** Pyrosequencing methylation levels identified across cg23703062. **(i)** Spearman correlation analysis of methylation levels from microarray and pyrosequencing analyses. **(j)** Methylation level across RFPL2 cg12906381 identified from microarray analysis. **(k)** Methylation level across cg12906381 from pyrosequencing analysis. **(l)** Spearman correlation of methylation levels across cg12906381 from microarray and pyrosequencing analyses. **(m)** Methylation level across RFPL2 cg01124132 from microarray analysis. **(n)** Methylation level across cg01124132 from pyrosequencing validation. **(o)** Spearman correlation between methylation levels identified from microarray and pyrosequencing analyses.

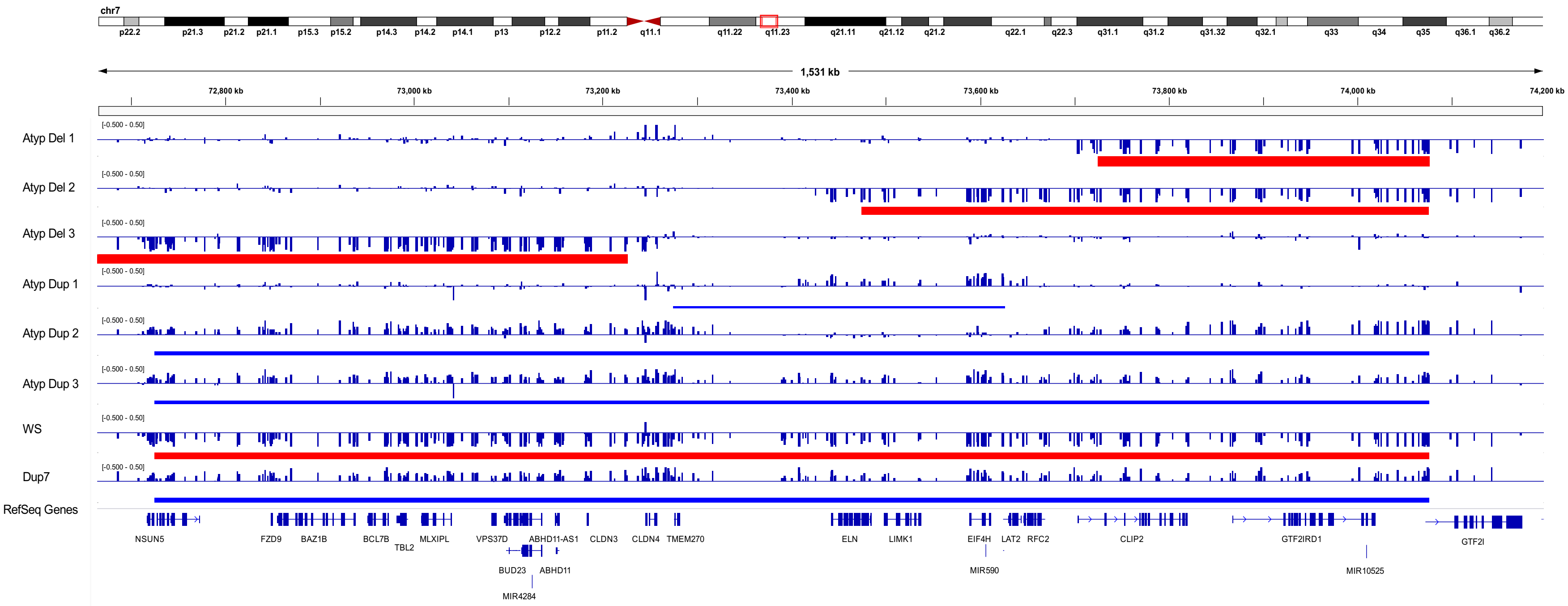

Supplementary Figure 4. Confirmation of 7q11.23 copy number using DNA methylation signals. Shown above is the output of Conumee[51] copy number variant analysis of each atypical participant, a typical 7q11.23 deletion (WBS) and a typical 7q11.23 duplication (Dup7) individual. Probes and their individually calculated copy number change are shown as blue vertical bars. The total region of copy number loss (red) or gain (blue) calculated by Conumee[51] is depicted as a horizontal bar beneath the probe signal. The height of the copy number segment is proportional to the average copy number change over the region, as calculated by Conumee[51].

Supplementary Table 1. ***GTF2I*** **expression in participants with atypical 7q11.23 CNV**

Gene	Participant	Genomic copy number	Relative expression
**GTF2I exon 3**	WBS	1 copy	0.54 +/- 0.10
	Dup7	3 copies	2.31 +/- 0.55
	Atyp Del 1	1 copy	0.51 +/- 0.08
	Atyp Del 2	1 copy	0.40 +/- 0.18
	Atyp Del 3	2 copies	0.84 +/- 0.04
	Atyp Dup 1	2 copies	1.08 +/- 0.29
	Atyp Dup 2	3 copies	n/a

**GTF2I exons 10-12**	Dup7	3 copies	1.74 +/- 0.52
	Atyp Dup 3	2 copies	1.30 +/- 0.35

Quantitative real-time PCR (qRT-PCR) was used to assess gene expression using primers listed in Supplementary Table 1. Expression values from each participant were normalized to the housekeeping genes *HMBS*, *HPRT* and *TBP* and are presented as average fold change relative to control participants with no 7q11.23 CNV. Relative expression of individuals with deletion (WBS) and duplication (Dup7) of 7q11.23 are shown for comparison. n/a – sample not available.

Supplementary Table 2. *BAZ1B*, *BCL7B* and *BUD23* expression in participants without CNV of these genes

Gene	Participant	Genomic DNA copy number	Relative expression
BAZ1B	WBS	1 copy	0.47 +/- 0.13
	Dup7	3 copies	1.98 +/- 0.41
	Atyp Del 1	2 copies	1.04 +/- 0.05
	Atyp Del 2	2 copies	0.81 +/- 0.33
	Atyp Dup 1	2 copies	1.18 +/- 0.39
BCL7B	WBS	1 copy	0.48 +/- 0.18
	Dup7	3 copies	2.68 +/- 0.17
	Atyp Del 1	2 copies	1.41 +/- 0.10
	Atyp Del 2	2 copies	0.97 +/- 0.39
	Atyp Dup 1	2 copies	1.12 +/- 0.45
BUD23	WBS	1 copy	0.51 +/- 0.24
	Dup7	3 copies	1.47 +/- 0.15
	Atyp Del 1	2 copies	0.89 +/- 0.29
	Atyp Del 2	2 copies	0.72 +/- 0.29
	Atyp Dup 1	2 copies	0.69 +/- 0.26

Quantitative real-time PCR (qRT-PCR) was used to assess gene expression using primers listed in Supplementary Table 1. Expression values from each participant were normalized to the housekeeping genes *HMBS*, *HPRT* and *TBP* and are presented as average fold change relative to control participants with no 7q11.23 CNV. Relative expression of individuals with deletion (WBS) and duplication (Dup7) of 7q11.23 are shown for comparison.

Supplementary Table 3. Gene expression primers

Gene	Primer	Sequence (5' to 3')
HMBS	Forward<br>Reverse	AGG CAT CAC TGC TCG TAA CA<br>GAT GTT TTT GGC TCC TTT GC
HPRT	Forward<br>Reverse	GCC TAT AGA CTA TCA GTT CCC TTT GG<br>TGC TGT GGT TTA AGA GAA TTT TTT CA
TBP	Forward<br>Reverse	GAT GCC TTA TGG CAC TGG AC<br>GCC TTT GTT GCT CTT CCA AA
BAZ1B	Forward<br>Reverse	ACA GGG TGT TGG TCA TCC TC<br>TGC AGA GTC AAG GGG ATT TC
BCL7B	Forward<br>Reverse	AGC AAC CAG AGT TCC GTG TC<br>GCT CAG GGA CTC ACT CTG CT
BUD23	Forward<br>Reverse	AGA CCT GAC ACC CAG TAC ACC<br>AGA AAA GTG CAG AGG CAA GTG
GTF2I exon 3	Forward<br>Reverse	GAA ATC TAC AAC CCA GGC AAA<br>GCA AAA GCA GAA ATA GTC CTC
GTF2I exon 10-12	Forward<br>Reverse	CAG GCC CTT CTG AAA CTG AT<br>CAG GGT CCT CAC TTG TTT CTG
