# Supplementary material for: DNA methylation profiles in individuals with rare, atypical 7q11.23 CNVs correlate with GTF2I and GTF2IRD1 copy number
Source: NPJ Genom Med. 2023 Sep 14;8:25. doi: 10.1038/s41525-023-00368-7 (PMC10502022; doi:10.1038/s41525-023-00368-7)
Supplement: Supplementary file 2 — Reporting Summary [file 41525_2023_368_MOESM2_ESM.pdf]

nature research

Corresponding author(s): Lucy R Osborne

Last updated by author(s): Nov 3, 2020

Reporting Summary

Nature Research wishes to improve the reproducibility of the work that we publish. This form provides structure for consistency and transparency in reporting. For further information on Nature Research policies, see our Editorial Policies and the Editorial Policy Checklist.

Statistics

For all statistical analyses, confirm that the following items are present in the figure legend, table legend, main text, or Methods section.

n/a	Confirmed	
☐	☒	The exact sample size (*n*) for each experimental group/condition, given as a discrete number and unit of measurement
☐	☒	A statement on whether measurements were taken from distinct samples or whether the same sample was measured repeatedly
☐	☒	The statistical test(s) used AND whether they are one- or two-sided<br>*Only common tests should be described solely by name; describe more complex techniques in the Methods section.*
☐	☒	A description of all covariates tested
☒	☐	A description of any assumptions or corrections, such as tests of normality and adjustment for multiple comparisons
☐	☒	A full description of the statistical parameters including central tendency (e.g. means) or other basic estimates (e.g. regression coefficient) AND variation (e.g. standard deviation) or associated estimates of uncertainty (e.g. confidence intervals)
☒	☐	For null hypothesis testing, the test statistic (e.g. *F*, *t*, *r*) with confidence intervals, effect sizes, degrees of freedom and *P* value noted<br>*Give P values as exact values whenever suitable.*
☒	☐	For Bayesian analysis, information on the choice of priors and Markov chain Monte Carlo settings
☒	☐	For hierarchical and complex designs, identification of the appropriate level for tests and full reporting of outcomes
☒	☐	Estimates of effect sizes (e.g. Cohen's *d*, Pearson's *r*), indicating how they were calculated

Our web collection on statistics for biologists contains articles on many of the points above.

Software and code

Policy information about availability of computer code

Data collection	Methylation data were collected using the HumanMethylation450k microarray, and primary image files collected using the iScan system from Illumina. Data from CNV analyses were collected from Clinical Diagnostic Laboratory reports and were independently confirmed using real time qPCR.
Data analysis	Methylation array image files were processed using the R package minfi version 1.24.0 and R version 3.1.3, with illumina-based normalization. Principal component analyses (PCA) and multidimensional scaling analysis (MDS) was performed using code available within the minfi data package. Correction for whole blood cell populations was performed by first estimating the proportion of each cell type using the estimateCellCounts function in minfi. When statistical analyses of significantly differentially methylated CpGs was performed, the cell type proportions derived from estimateCellCounts, the sex and age of participants were used as covariates in a linear regression model using Limma version 3.34.9. As a secondary control for sample mix-up, CNV calls were derived from the methylation data using the R package conumee v1.4.2. Estimated CNVs from conumee were plotted using igv software v2.8.2. Hierarchical clustering and heatmap analysis was performed using the R stats package (version 3.4.4) function hclust (euclidean distance matrix and ward clustering method). Correlation analyses of pyrosequencing and array based data were performed using the R stats package (version 3.4.4) function 'cor' with employing Spearman correlation analyses.

For manuscripts utilizing custom algorithms or software that are central to the research but not yet described in published literature, software must be made available to editors and reviewers. We strongly encourage code deposition in a community repository (e.g. GitHub). See the Nature Research guidelines for submitting code & software for further information.

April 2020

Data

Policy information about availability of data

All manuscripts must include a data availability statement. This statement should provide the following information, where applicable:

- Accession codes, unique identifiers, or web links for publicly available datasets
- A list of figures that have associated raw data
- A description of any restrictions on data availability

Raw methylation data will be deposited into the GEO data repository prior to publication. Figures 3 and 4 contain DNA methylation data analysed from the associated raw data files.

Field-specific reporting

Please select the one below that is the best fit for your research. If you are not sure, read the appropriate sections before making your selection.

☒ Life sciences ☐ Behavioural & social sciences ☐ Ecological, evolutionary & environmental sciences

For a reference copy of the document with all sections, see nature.com/documents/nr-reporting-summary-flat.pdf

Life sciences study design

All studies must disclose on these points even when the disclosure is negative.

Sample size	Atypical samples were collected when recruited into the larger research study program. Given the rarity of these individuals, only very few atypical participants were available to us and consented to participate in our study. In addition, because each atypical participant is genotypically distinct they are treated as individuals, not as a population.
Data exclusions	After verifying that our data did not contain significant batch effects or technical variation, the technical replicate samples were excluded from downstream analyses to protect against artificial skewing of the data, given our small sample size.
Replication	Previously published results of whole genome methylation analyses from our group were independently validated in this study by re-running the entire analysis from the raw data files. This analysis contained the additional atypical participants, and by including 4 technical replicates (one from each genotypic group), we both replicated our original findings and performed internal technical replication to monitor for batch effects and other technical variables.
Randomization	Randomization was not relevant to this study as we were performing a genotype-driven analysis of DNA methylation. Applicable covariates were proportions of whole blood cell populations, sex and age of participants, which are known to skew methylation data. When required for analyses of differential methylation, these variables were incorporated as covariates in the R package Limma.
Blinding	Investigators were blinded to the allocation of each sample to a specific genotype group at the point of DNA methylation data collection (methylation array). Overall quality control, performance metrics and initial data analyses using MDS and PCA were performed independent of group allocation.

Reporting for specific materials, systems and methods

We require information from authors about some types of materials, experimental systems and methods used in many studies. Here, indicate whether each material, system or method listed is relevant to your study. If you are not sure if a list item applies to your research, read the appropriate section before selecting a response.

Materials & experimental systems

n/a	Involved in the study	
☒	☐	Antibodies
☒	☐	Eukaryotic cell lines
☒	☐	Palaeontology and archaeology
☒	☐	Animals and other organisms
☐	☒	Human research participants
☐	☒	Clinical data
☒	☐	Dual use research of concern

Methods

n/a	Involved in the study	
☒	☐	ChIP-seq
☒	☐	Flow cytometry
☒	☐	MRI-based neuroimaging

Human research participants

Policy information about studies involving human research participants

Population characteristics	Atypical deletion and duplication participants were children (age range 4.12 to 11.93 years of age). Four were female and two

Population characteristics	were male. Participants in the Williams syndrome, 7q11.23 duplication syndrome, and typically developing control groups were also children and have been reported previously (Strong et al. AJHG 2015). Due to the uniqueness of the genetic rearrangements in each of the atypical participants, they were not treated as a population but as distinct individuals. Individual clinical characteristics are included in the Results section and individual genetic characteristics are reported in the Results section and in Table 1.
Recruitment	Participants were recruited through referral from clinical genetics departments at various hospitals. All had previously undergone clinical microarray analysis that showed an atypical deletion or duplication of the 7q11.23 region.
Ethics oversight	Research Ethics Board of the University of Toronto, Institutional Review Board of the University of Nevada, Reno and Institutional Review Board of the University of Louisville.

Note that full information on the approval of the study protocol must also be provided in the manuscript.

Clinical data

Policy information about clinical studies

All manuscripts should comply with the ICMJE guidelines for publication of clinical research and a completed CONSORT checklist must be included with all submissions.

Clinical trial registration	*Provide the trial registration number from ClinicalTrials.gov or an equivalent agency.*
Study protocol	*Note where the full trial protocol can be accessed OR if not available, explain why.*
Data collection	*Describe the settings and locales of data collection, noting the time periods of recruitment and data collection.*
Outcomes	*Describe how you pre-defined primary and secondary outcome measures and how you assessed these measures.*
